# Evaluating regional blood spinal cord barrier dysfunction following spinal cord injury using longitudinal dynamic contrast-enhanced MRI

**DOI:** 10.1186/1471-2342-9-10

**Published:** 2009-06-11

**Authors:** Ilkan Tatar, Peter Cheng-te Chou, Mohamed Mokhtar Desouki, Hanaa El Sayed, Mehmet Bilgen

**Affiliations:** 1 Preclinical Imaging in Translational Research Laboratory, Radiology and Radiological Science, Medical University of South Carolina, 169 Ashley Avenue, Charleston, SC 29425, USA; 2 Pathology, Medical University of South Carolina, 169 Ashley Avenue, Charleston, SC 29425, USA; 3 Neuroscience, Medical University of South Carolina, 169 Ashley Avenue, Charleston, SC 29425, USA

## Abstract

**Background:**

In vivo preclinical imaging of spinal cord injury (SCI) in rodent models provides clinically relevant information in translational research. This paper uses multimodal magnetic resonance imaging (MRI) to investigate neurovascular pathology and changes in blood spinal cord barrier (BSCB) permeability following SCI in a mouse model of SCI.

**Methods:**

C57BL/6 female mice (n = 5) were subjected to contusive injury at the thoracic T11 level and scanned on post injury days 1 and 3 using anatomical, dynamic contrast-enhanced (DCE-MRI) and diffusion tensor imaging (DTI). The injured cords were evaluated postmortem with histopathological stains specific to neurovascular changes. A computational model was implemented to map local changes in barrier function from the contrast enhancement. The area and volume of spinal cord tissue with dysfunctional barrier were determined using semi-automatic segmentation.

**Results:**

Quantitative maps derived from the acquired DCE-MRI data depicted the degree of BSCB permeability variations in injured spinal cords. At the injury sites, the damaged barriers occupied about 70% of the total cross section and 48% of the total volume on day 1, but the corresponding measurements were reduced to 55% and 25%, respectively on day 3. These changes implied spatio-temporal remodeling of microvasculature and its architecture in injured SC. Diffusion computations included longitudinal and transverse diffusivities and fractional anisotropy index. Comparison of permeability and diffusion measurements indicated regions of injured cords with dysfunctional barriers had structural changes in the form of greater axonal loss and demyelination, as supported by histopathologic assessments.

**Conclusion:**

The results from this study collectively demonstrated the feasibility of quantitatively mapping regional BSCB dysfunction in injured cord in mouse and obtaining complementary information about its structural integrity using in vivo DCE-MRI and DTI protocols. This capability is expected to play an important role in characterizing the neurovascular changes and reorganization following SCI in longitudinal preclinical experiments, but with potential clinical implications.

## Background

Damage to blood-spinal cord barrier (BSCB) occurs as a consequence of mechanical insult to spinal cord (SC) [1]. The damaged barrier becomes permeable to blood constituents, inflammatory cells and other large molecules, which collectively activate a cascade of secondary processes harmful to the underlying tissue. With time, vascular bed of the injured SC goes through repair and remodeling [2]. Providing nutrition and preventing extravasation of destructive biochemical compounds in blood protect the remaining neurons and maintain the existing substrate from further degeneration. At the same time, knowledge of barrier properties and its status is essential if potential intravascular drugs with capabilities to improve neurovascular protection and to promote repair and recovery will be administered. Such treatment is possible provided that the drugs can pass through the open barriers to reach the destined regions in the SC parenchyma. Therefore, a complete understanding of the vascular response to spinal cord injury (SCI) is required for developing intervention strategies aimed at rapidly restoring the barrier integrity as well as blood supply to the ischemic areas of the traumatized cord.

So far, BSCB permeability changes in injured SC have been evaluated using a range of tracers and laborious postmortem tissue analysis [3]. Alternatively, in vivo dynamic contrast-enhanced MRI (DCE-MRI) was proposed to noninvasively visualize and quantify the changes in BSCB permeability in a relatively more efficient way [4-6]. The initial DCE-MRI studies were performed using a rat animal model of SCI. Lately, the availability of diverse strains and transgenic varieties has made the mouse a more attractive model [7-10]. But, to date, DCE-MRI studies on mouse are lacking. The first goal of this paper is to demonstrate the feasibility of performing DCE-MRI on mouse with SCI.

The contrast enhanced data in previous rat studies were acquired dynamically over an extended period of time and processed using a pharmacokinetic model with complicated numerical computation routine. The end results from the computation included quantitative measurements that represented the overall exchange of contrast agent between plasma and lesion. Such representation was useful, but yielded limited information, since in practice, rather than global evaluation, more detailed local variations in BSCB permeability was sought after. In this previous approach, the DCE-MRI data acquisition time was long since it required covering both the wash-in and wash-out phases of the contrast agent. Another weakness was that the parameters were estimated using a time-inefficient computation algorithm. Thus, the second goal of this study is to provide an improved method in terms of data collection, post processing and spatial representation of the BSCB permeability variations in injured SC.

Histological studies have demonstrated that, in injured SC, the areas with damaged vasculature overlap with the areas of intense neuronal loss [11,12]. Noninvasive diffusion tensor imaging (DTI) provides information about the neuronal integrity in underlying SC tissue [13,14]. Combining these, it is likely that in vivo BSCB permeability and DTI-based measurements, both obtained from the same region of the injured SC, can provide relevant information about the viability of underlying neurovasculature, which has so far only been available by performing ex vivo histological analysis. Therefore, the third goal of this study is to compare the BSCB permeability and DTI measurements and investigate the nature of their association while assessing the neurovascular response in SCI.

Studies based on postmortem tissue analysis suggest that the first 72 hours following SCI are the most critical, offering a window of opportunity for potential treatments [15-18]. In order to develop effective therapeutic strategies in experimental studies, it is important to employ a SCI model that sensitively responds to the treatment at the acute phase of the injury and is also capable of producing measurable vascular and neuropathological changes within the first 72 hr time frame. Therefore, this study focused on the acute phase (postinjury days 1 and 3) of the injury with the above pathological properties and investigated the injured cords using multimodal neuroimaging (anatomical, DCE-MRI and DTI) noninvasively. In the following, we first describe our imaging protocols, and then give details of our implementation and basis of our data processing algorithm and strategies. Next, using these developments, we present results from neuropathological evaluations and generate reliable BSCB permeability maps to show the spatiotemporal course of the alterations in vascular permeability within the SC lesion and its surroundings. We characterize the area and volume of the regions with dysfunctional BSCB. We also test the BSCB permeability estimates against the DTI measurements from the corresponding regions to establish the level of association as determined from statistical analysis.

## Methods

All experiments were carried out with twelve-week old female C57BL/6 mice (n = 5) in accordance with a protocol approved by the Institutional Animal Care and Use Committee. All of the mice were subjected to SCI and participated in the MRI scans using DCE-MRI and DTI protocols on postinjury days 1 and 3.

### Procedures for surgeries and spinal cord injury

All surgeries were performed in sterile conditions. The mouse was initially anesthetized by a spontaneous inhalation of 4% isoflurane in an induction chamber and then moved to a surgery mat. The anesthesia level was reduced to 2% isoflurane delivered in a mixture of 40% oxygen and 58% air through a nose mask. Further adjustments in small increments were made on the percentage of the isoflurane level during the surgery. For intravascular delivery of the paramagnetic contrast agent with MW = 938 Da (Magnevist, Berlex Imaging, Wayne, NJ) in DCE-MRI studies, animals underwent additional jugular vein catheterization with PE-10 tubing [5]. The catheter ran subcutaneously and exited the skin at the back between shoulder blades, then was kept folded behind the animal. The catheter was flushed with heparin daily to prevent clotting. The surgical procedures for inducing SCI involved a midline incision posterior from the thoracic levels T10 to T12, followed by dissection of the bilateral vertebral muscles to expose the dorsal laminae and spinous processes. Laminectomy was performed at the T11 level to expose the SC by exercising special care not to damage the dura mater. The spinous processes at T10 and T12 adjacent to the laminectomy were stabilized using two hemostatic forceps. A 1-mm diameter injury bit, that was attached to a generic central nervous system injury device described earlier [19], was positioned perpendicular to the dorsal surface of the SC. The device consists of electromechanical components – a linear motor connected to a controller. The controller communicated with a personal computer through a software program developed in our laboratory to input biomechanical parameter values for inducing a contusion-type SCI. The injury parameters used in these experiments were: impact velocity of 0.75 m/s; surface displacement depth of 0.5 mm; and compression duration of 85 ms. After the injury, the overlying muscle layers were sutured and skin was closed tightly. Then, the injured mouse was left to recover in a heated cage and received postoperative care.

### MRI scans

Each injured mouse was scanned on days 1 and 3 using a 9.4 T horizontal INOVA Varian system (Varian, Palo Alto, CA) and an inductively coupled surface coil [20]. The scan was performed when the mouse was under a general anesthesia which was delivered as a mixture of 2% isoflurane, 40% oxygen and 58% air through a nose mask. Vital signs (respiration, heart rate and body temperature) of the anesthetized animal were monitored using a MRI-compatible monitoring and gating system (Model 1025, SA Instruments Inc., Stony Brook, NY). Respiratory-gated acquisition was used to increase the image quality by minimizing breathing related image artifacts.

High-resolution anatomical images on all animals were first acquired in sagittal and axial views using a spin-echo sequence in multislice and interleaved fashion. The scan parameters for the sagittal or horizontal images were TR/TE = 2500/12 ms, field-of-view (FOV) = 26 × 8 mm^2^, matrix = 256 × 128, in-plane pixel resolution = 100 × 63 μm, slice thickness = 0.5 mm, number of excitations (NEX) = 2. The corresponding parameters for the axial images were TR/TE = 2500/12 ms, FOV = 12 × 8 mm^2^, matrix = 128 × 128, in-plane pixel resolution = 94 × 63 μm, slice thickness = 1 mm, number of slices = 14 and NEX = 2. These data constituted the proton density (PD) weighted images.

Then, for microstructural imaging, diffusion weighted images were acquired using diffusion gradient strength = 80 mT/m, width (δ) = 6.5 ms and separation (Δ) = 11 ms to produce a b-value of 534 s/mm^2^. The imaging parameters for these scans were TR/TE = 2000/26 ms, FOV = 12 × 8 mm^2^, image matrix = 128 × 128, in-plane pixel resolution = 94 × 63 μm, slice thickness = 1 mm and NEX = 2 [20-22]. Baseline data acquired in the absence of the diffusion weighting constituted the T2-weighted images.

Next, for evaluating microvascular response and imaging BSCB permeability to the contrast agent in the injured animal, a DCE-MRI protocol was applied [5]. The contrast agent was delivered as a bolus (< 5 s) at a dose of 0.1 mmol/kg while the animal was still in the scanner. To detect the contrast enhancement, T1-weighted axial images were acquired precontrast and repetitively for up to 2 hr postcontrast using the same parameters as the PD weighted images but with TR = 1000 ms and NEX = 4. The temporal resolution between these acquisitions was 10 min.

### Quantitative MRI data analysis

The MRI data were acquired and visualized using the scanner's control software VNMRJ (Varian, Palo Alto, CA).

#### DCE-MRI Analysis

The DCE-MRI data were analyzed off-line using custom-written software in Matlab (The Mathworks, Inc., Natick, MA). Axial images acquired before and after the contrast enhancement in a given scanning session were loaded into computer and processed interactively using a graphical user interface. The details of the performed numerical analysis are described in Appendix. The results are 2-D maps that quantitatively describe the BSCB permeability (denoted by the parameter *K*_*p*-*sc*_) throughout the injured SC.

### Area and volume of dysfunctional BSCB in injured SC

By analyzing the spatial distribution of the permeability changes on *K*_*p*-*sc *_maps, we measured the area and volume of the injured SC tissue with damaged BSCB. To perform the area measurements, a threshold value was required to differentiate the elevated *K*_*p*-*sc *_values from the background noise. The value of this threshold was determined while postprocessing the data. To accomplish this task, an algorithm was implemented using the theory of signal detection in the presence of additive noise. The procedure involved first computing histograms of *K*_*p*-*sc *_maps from two normal sections of the injured cord, but within the masks of the corresponding slices at 4 mm caudal and rostral to the injury epicenter. Because BSCB in these regions were intact, the contrast agent was retained within the SC vasculature and no detectable leakage took place. These histograms depicted *K*_*p*-*sc *_estimates that were normally distributed around zero, which could be fitted to zero mean Gaussian profiles (please see the results section). The larger standard deviation *σ *from the two Gaussian fits (from a caudal and a rostral slice) was used to set a threshold value for *K*_*p*-*sc *_at +2*σ *. This selection was based on the property that the Gaussian distribution between -2*σ *and +2*σ *equals to nearly 95% of the total area beneath the curve. This meant that nearly 97.5% of the pixels in the *K*_*p*-*sc *_map from a normal SC were below the threshold and therefore considered to have intact BSCB. The same threshold value was employed for analyzing the *K*_*p*-*sc *_maps produced for all the remaining slices. The histograms from slices near the injury epicenter were observed to be shifted towards higher *K*_*p*-*sc *_values, as expected, reflecting the presence of compromised BSCB. These histograms had wider spread but still followed the profile of Gaussian distribution.

The number of pixels whose intensity values on the *K*_*p*-*sc *_maps remained above the threshold was counted to determine the area of the SC with compromised BSCB in a given slice. This number was further divided by the total cross sectional area of the cord in that slice to obtain a measure of normalized area (*NA*). The slice with largest pixel count was considered as representing the injury epicenter. The volume of the compromised BSCB was determined by summing the areas (prior to normalization) in six neighboring slices covering the epicenter. Total cord volume was similarly calculated from the total cord area in each slice. The total volume with compromised BSCB was scaled by the total SC volume to obtain normalized volume (*NV*). This normalization of the area and volume measurements compensated for the variations from slice to slice and animal to animal, as well as other spatial scales, such as changes in slice thickness and pixel dimensions.

In addition, *K*_*p*-*sc *_measurements remaining above threshold within the total area at the epicenter slice were averaged for each animal. The resulting averages for day 1 and day 3 were then correlated with the corresponding mean DTI measurements within the same area, as explained below.

### DTI analysis

Diffusion-weighted images were processed and the elements of diffusion tensor were estimated for each image voxel using the scanner software VNMRJ (Varian Inc., Palo Alto, CA). The diffusion tensor represents the statistical distribution of microscopic motion of water molecules, and its three eigenvalues (λ_1_, λ_2 _and λ_3_) characterize the principal water diffusivities along its three orthogonal eigenvectors [21,23,24]. To be consistent with the nomenclature used in previous reports, we denoted λ_1 _= λ_|| _as an expression of longitudinal diffusivity of water molecules along the axonal fibers, and λ_⊥ _= (λ_2_+λ_3_)/2 as an expression of transverse diffusivity for water moving perpendicular to the direction of neuronal fibers in SC. The fractional anisotropy (*FA*) index was computed algebraically by combining the eigenvalues. FA index is a rotationally invariant scalar and quantitatively characterizes the degree of anisotropy in the diffusion properties of the underlying tissue within the voxel. The parameters λ_1_, λ_⊥ _and *FA *were estimated for each voxel throughout the slice at the epicenter and the corresponding maps were generated in 2-D. Similar to computing mean *K*_*p*-*sc *_values described above, λ_||_, λ_⊥ _and FA measurements from the areas with compromised BSCB at the epicenter were averaged and the resulting mean values for each animal were recorded in a database along with the corresponding mean *K*_*p*-*sc*_. Group mean and standard deviation of these measurements were again computed for days 1 and 3 separately.

### Postmortem tissue analysis

Neurovascular histopathologies of selected injured SCs were examined postmortem following MRI scan on day 3. Each mouse was euthanized by intracardiac perfusion with 50 mL of phosphate buffered saline (PBS) solution, followed by 50 mL of 4% formaldehyde PBS solution that were delivered through a 23-gauge needle connected to a perfusion pump. The SC was excised and fixed in 4% formaldehyde. Segments from the injury epicenter or normal levels were embedded in paraffin and cut serially in 10 μm thick sections. Representative samples were stained with standard hematoxylin and eosin (H&E), luxol fast blue (LFB), vascular endothelial marker (CD34) or neuron specific enolase (NSE) for histopathological assessment of the neurovascular pathological changes. Immunohistochemistry (IHC) was carried out with CD34 (MY 10 clone, BD Bioscience, Franklin Lakes, NJ) (1:100 dilution) and NSE (BBS/NC/VI-H14 clone, Dako Cytomation, Carpinteria, CA) antibodies using a Dako cytomation autostainer [25]. Briefly, the slides were de-paraffinized by incubation in xylene and ascending grades of alcohol. Antigen retrieval for CD34 was done by incubation with proteinase Kinase at room temperature for 5 minutes. Antigen retrieval for NSE was done by heating in ethylene diamine tetraacetic acid (EDTA), PH 9.0 buffer (Lab vision, Fremont, CA) at 95°C for 20 min. Both sections were loaded in the autostainer programmed as follows: 3% hydrogen peroxide for 10 minutes, blocked with 5% skimmed milk for 5 min, incubated with primary antibody for 30 min, followed by incubation with Envision+ system HRP (Dako Cytomation, Carpinteria, CA) for 30 minutes. Color was developed by incubating samples with diaminobenzidine (DAB)+chromogin for 10 min followed by Dako DAB enhancer for 5 min. Hematoxylin was used as counter stain. The sections were examined using a BX 50 Olympus microscope and the photographs were taken with an attached Olympus DP 70 camera operated with DP Controller software (Olympus Corporation, Center Valley, PA). One section was also incubated with secondary antibody only to check nonspecific binding.

### Statistical analysis

The quantitative data collected from the measurements were analyzed statistically. The *NA*, *NV*, *K*_*p*-*sc*_, λ_||_, λ_⊥ _and *FA *values gathered from each animal were listed in two groups as day 1 and day 3, and the means and standard deviations within each group were computed for each parameter to understand the intra-group variations. The measurements between day 1 and 3 were compared using the paired Student's *t *test. This analysis allowed examination of inter-group variations and the determination of the statistical significance of the differences. Statistical significance was defined at P < 0.05. Also, statistical dependencies between *K*_*p*-*sc*_, and measurements λ_||_, λ_⊥ _and *FA *were determined using Pearson's correlation analysis and the resulting correlation coefficients were reported.

## Results

The surgical procedures, injuries, and prolonged anesthesia during MRI scans were well tolerated by all mice. For imaging, each mouse was carefully placed supine over an inductively coupled radio frequency coil system to achieve an optimal tuning and matching condition as observed in the frequency response of the coil impedance. When the animal was placed in the magnet, the catheter attached to it was extended outside at the front end of the magnet bore. The injured cord was then imaged in transverse and rostral-caudal planes. Acquiring data from different orientations allowed better evaluation of the lesion's spatial extent.

**Figure 1:**
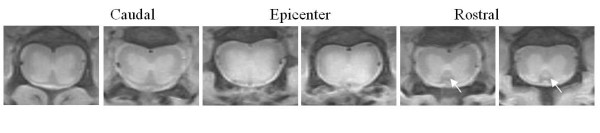
**Axial proton-density images of an injured mouse spinal cord**. The serial images show normal caudal and rostral sections and injury epicenter on postinjury day 1. Arrows point to corticospinal tract (CST). In normal cord, the image intensity profile does not produce enough contrast to distinguish the CST from the surrounding white matter. Interestingly, in this injured SC, the CST at the rostral section, but not the caudal section, has been delineated by slight hyperintensity compared to the background white matter.

Anatomical axial PD images acquired from one of the injured SC are shown in Figure 1. The intensity contrasts on these images from normal sections delineate gross anatomical details of the cord within the white matter (WM) and grey matter (GM) as well as the surrounding spinal structures. The lesion is depicted by an altered intensity contrast in the parenchyma below the laminectomy. The corticospinal tract in the mouse is anatomically located between the dorsal horns ventrally next to the central canal. In normal cords, the image intensity profile does not produce enough contrast to distinguish this tract from the surrounding WM and GM. In this particular injured SC, images from the rostral, but not the caudal, sections delineated the corticospinal tract with hyperintensity. The intensity change was indicative of alterations in the MR properties of the tract and of a pathological abnormality, which was likely associated with Wallerian degeneration [26].

**Figure 2:**
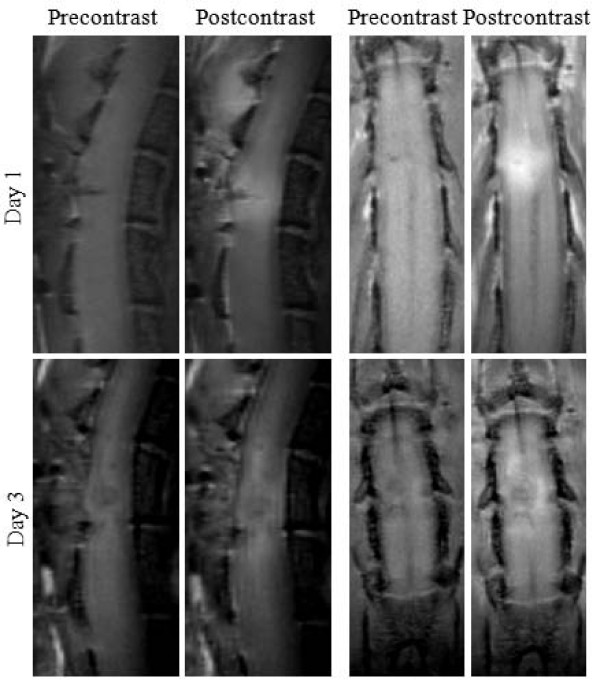
**Precontrast and postcontrast T1-weighted images of injured mouse spinal cord in sagittal and horizontal views**. The postcontrast images were acquired 130 min (sagittal) and 140 min (horizontal) after the IV bolus delivery of the contrast agent.

Figure 2 presents pre- and postcontrast T1-weighted images in sagittal and horizontal planes in both postinjury days 1 and 3. On day 1, the precontrast images revealed a small focal hypointensity close to the dorsal surface reflective of neuropathology. On day 3, the lesion assumed a circular shape and enlarged in size. The postcontrast images depicted intensity enhancement at the lesion and its surroundings. The hyperintense regions in postcontrast images represented areas of injured SC tissue loaded with the contrast agent due to its leakage through the compromised BSCB therein.

The postcontrast sagittal images in the figure were acquired 130 min and those in the horizontal views were acquired 140 min after the delivery of the contrast agent. Following its delivery, the contrast agent diffuses passively in the extravascular spaces of the injured SC parenchyma. This leads to the brightness enhancement spreading along the cord in both rostral and caudal directions, as evident in the figure.

**Figure 3:**
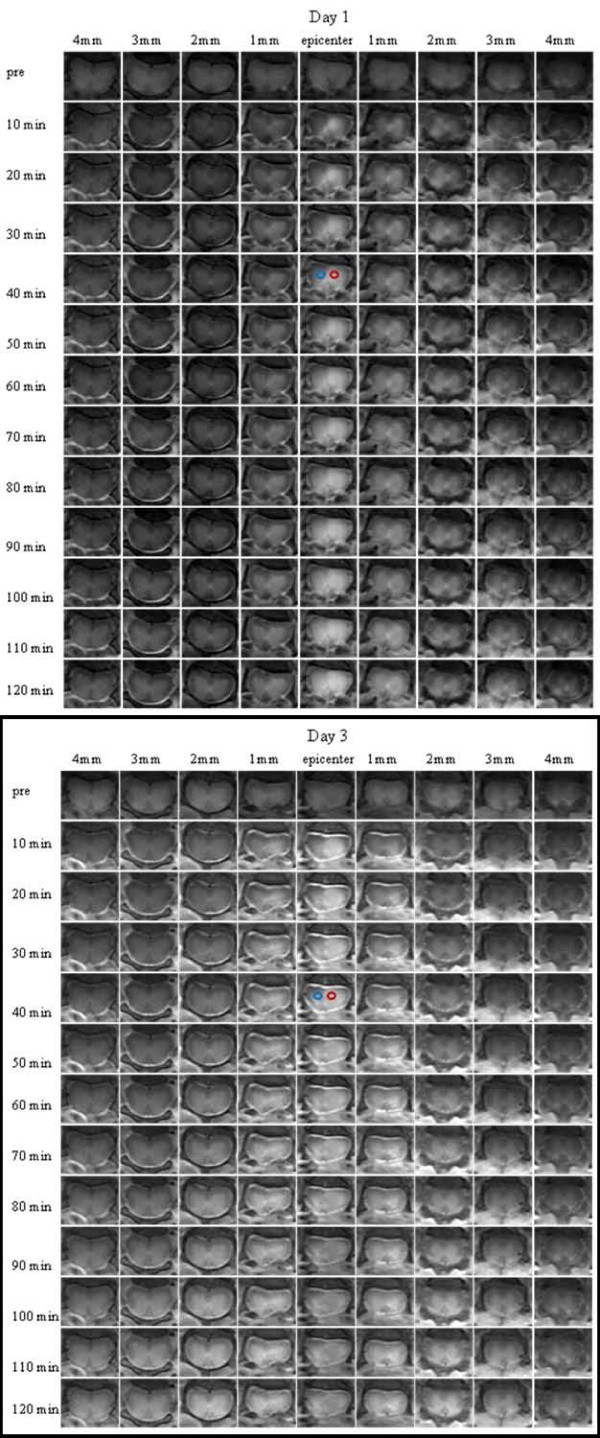
**Contrast enhancement following the administration of the contrast agent with time and space on postinjury day 1 and day 3**. The red and blue circles represent the regions of interest selected to produce the data in Figure 4. Please note that intensity enhancement induced by the presence of the contrast agent in cerebrospinal fluid.

The brightness enhancement expanding spatially with time was better appreciated in images from axial views in Figure 3. This figure shows strong contrast enhancement early on at the lesion and its immediate surroundings following the contrast agent delivery. The enhancement was mostly localized in the GM than the WM. But as time progressed, the SC parenchyma became brighter at the slices distant from the epicenter in both caudal and rostral directions within the normal sections of the injured SC. Although the patterns of contrast enhancements on days 1 and 3 were similar for this exam, the data indicated that the enhancement on day 1 peaked at the epicenter and skewed asymmetrically towards the rostral direction, unlike more symmetric, but wider, enhancement along the cord as visualized on day 3. In addition, cerebrospinal fluid (CSF) appeared hyperintense on the postcontrast images. This was another important observation, which was also reported in the previous DCE-MRI study with a rat model of SCI [5]. Combining the CSF enhancement in rat with the current observation made in mouse, it is likely that rodents may have a unique system of CSF circulation different than human, since CSF of human does not typically enhance after the IV delivery of the contrast agent used in this study.

For the data analysis, images (precontrast and first postcontrast) from a given slice location were displayed simultaneously. Image alignment was verified visually by zooming in on anatomical landmarks. Two out of the ten DCE-MRI scans exhibited spatial misalignment between the axial postcontrast and the corresponding precontrast images. In these cases, the sedated mouse reacts to the contrast agent, causing its body to move slightly. From the evaluation of the images, it was evident that the image motions in the misaligned data sets were translational in both cases, but a slight rotation was present in one of them. Neither of the motions was of deformation type. The two misaligned images were registered using a simple postprocessing technique with Matlab's "circshift" and "rotate" functions. A more complicated automated algorithm for image registration chould have been used for the registration. But this required complex implementation, which was beyond the scope of the study.

**Figure 4:**
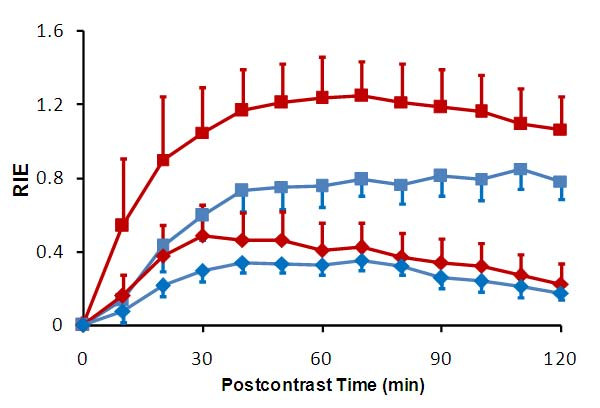
**Temporal patterns of relative intensity enhancement (*RIE*) averaged within two selected regions of interest (red and blue circles in Figure 5) and on Day 1 (square) and Day 3 (diamond)**.

Figure 4 compares the temporal profile of the relative intensity enhancement (*REI*, computed using Eq. 1 in the Appendix) within the regions of interest at the injury site on both days 1 and 3. The selected regions were identified by circles coded with two different colors in Figure 3. The variations in plots with different colors on the same day indicated *REI *has spatial dependence. The identical colors showed that the average *REI *profiles varied between day 1 and day 3. According to Eq. 2, *REI *reflected the amount of localized accumulation of the contrast agent, and thereby indicated the severity of leakage of the agent through the dysfunctional BSCBs therein.

**Figure 5:**
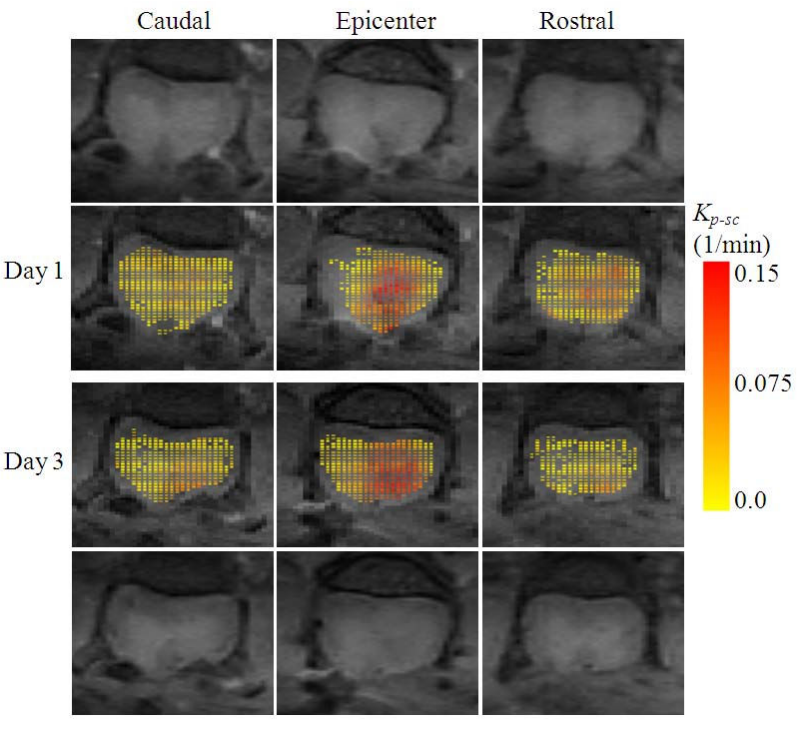
**Color coded *K*_*p*-*sc*_-maps computed using Eq. 4 from the three slices centered at the epicenter using the data set in Figure 4**. The backgrounds are the T1-weighted precontrast images (top and bottom rows). The color from yellow to red indicates linearly increasing compromise in blood spinal cord barrier permeability. The areas with color towards red indicate regions with more vascular damage causing the barriers to become more permeable to blood constituents.

Figure 5 shows quantitative distribution of BSCB permeability for the data set in Figure 3. The *K*_*p*-*sc*_-maps in the figure were computed from three slices centered at the epicenter using Eq. 4 in Appendix. The permeability estimates were overlaid as a new layer with color coding on the precontrast T1-weighted images, which served as the background. In the maps, color coding towards red meant increased BSCB dysfunction. The red zones represented the regions with greater leakage of the contrast agent and overlapped consistently with the lesion pathology.

**Figure 6:**
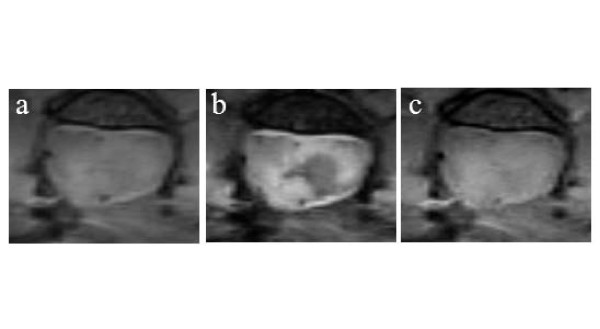
**Anatomical images of injury epicenter on post-injury day 3 prior to the injection of contrast agent**: a) PD image, b) T2-weighted image and c) T1-weighted image.

Figure 6 shows the precontrast PD and T2-weighted images of the same injured SC. The corresponding T1-weighted image was reproduced from Figure 3 for a side-by-side comparison. Depending on the acquisition protocol employed, images delineated the injury pathology with a specific intensity contrast. For this case, almost half of the GM appeared hypointense on the T2-weighted image (Figure 6-b), but the remaining contra-lateral section exhibited nearly homogeneous contrast, making it difficult to differentiate GM from WM. In addition, a thin strip of hyperintensity was observed along the circumference of the hypointense region on the T2-weighted image. In general, hypointensity on T2-weighted images in the early phase of SCI in mouse is associated with the accumulation of red blood cells, most likely escaping the vasculature that was ruptured by the initial mechanical impact. Hyperintensity is associated with vasogenic edema describing the accumulation of fluid containing plasma proteins into the extracellular spaces of the injured SC. The origin of these contrasts was determined by examining the corresponding histology slides.

**Figure 7:**
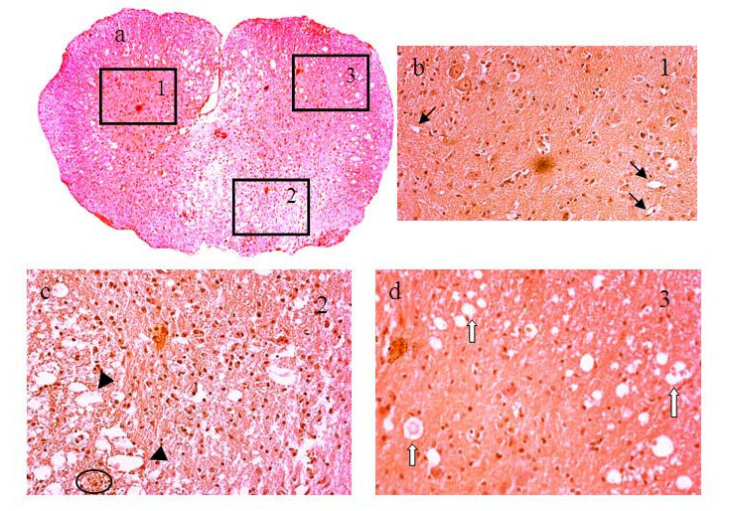
**H&E stained histology images of injured SC on day 3**: a) at lower magnification and b-d) at higher magnification within the selected square windows marked with numbers 1, 2 and 3 in panel a. The windows were selected based on the MRI-observed pathology in Figure 6. The 1^st ^window was selected in an intact GM region, the 2^nd ^window was selected in a significantly damaged region and the 3^rd ^window was selected in a region with edema. Black arrows denote normal vessels and capillaries. Black arrow heads point to disrupted vasculature with damaged BSCB. White arrows point to vessels surrounded by cavities. Circle denotes clusters of extravasated red blood cells.

**Figure 8:**
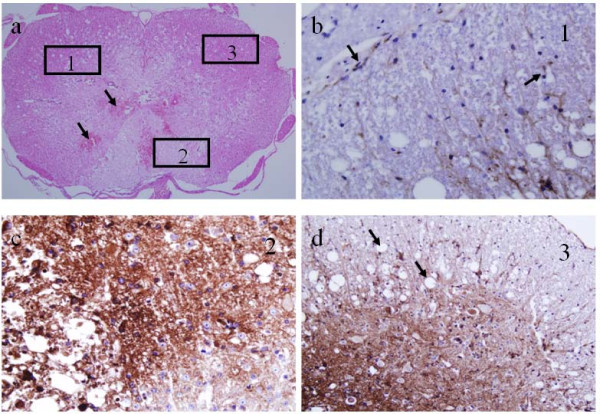
**Histology images of an injured SC on day 3**: a) H&E-stained section with arrows denotes extravasated red blood cells. b-d) higher magnifications of adjacent section stained with the endothelial marker, CD34. The square windows marked with numbers 1, 2 and 3 were selected to show vasculature and BSCB integrity therein. b) Sparse, intact vasculature (arrows). c) Disrupted vasculature with diffusion of immunostaining due to damaged BSCB. d) Disrupted vessels and tissue vacuolization due to edema from the leaky BSCB (arrows).

**Figure 9:**
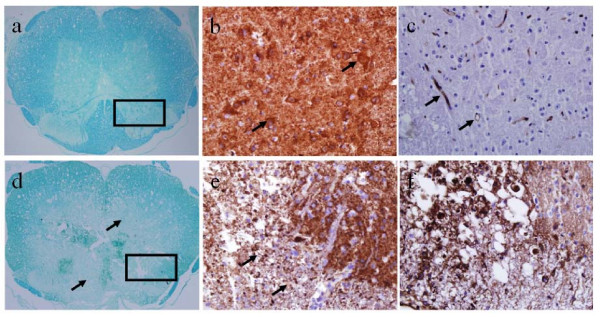
**LFB, NSE and CD34 stained contiguous slices at the injury epicenter and at a normal section rostral to the epicenter**. In the rostral segment (top row), LFB (a) and NSE (b) stains show normal myelination and intact neurons, and CD34 stain (c) shows intact vasculature. At the epicenter (bottom row), LFB (d) and NSE (e) stains show diffuse demyelination and damaged neurons, and the corresponding CD34 stain (f) shows damaged vasculature with leaky BSCB.

Figures 7, 8 and 9 show histologic sections stained with H&E, LFB, NSE or CD34. The slice in Figure 7 matches the images in Figure 6, but the others in Figures 8 and 9 are from a different injured SC. The vasculature was depicted by H&E staining in Figure 7 and better outlined in Figure 8 by CD34 immunostaining. The microscopic features of the neurovascular pathology revealed partially intact GM at the ventral horns, significant damage at the dorsal SC with substantial loss of tissue matrix and also small cavities distributed throughout the cord, but generally more prominent in WM than GM. These regions were evaluated more closely at higher magnification in three selected regions of interest – windows 1, 2 and 3. As expected, in the normal looking GM (window 1), the vessels were observed to be intact but mostly ruptured in the damaged region (window 2) that led to the extravasation of the red blood cells. The periphery of the damaged SC region (window 3) contained cavities that colocalized with small vessels or endothelial cells.

Figure 9 shows LFB, NSE and CD34 stained slices showing the injury and a normal cord section. At the injury, the LFB and NSE stains depicted diffuse demyelination and completely disorganized neurons and processes. The CD34 stain from the same areas showed damaged vasculature with leaky barrier. In the rostral segment, however, LFB and NSE stains indicated normal myelination and intact neurons, and CD34 stain showed vasculature with intact barrier.

**Figure 10:**
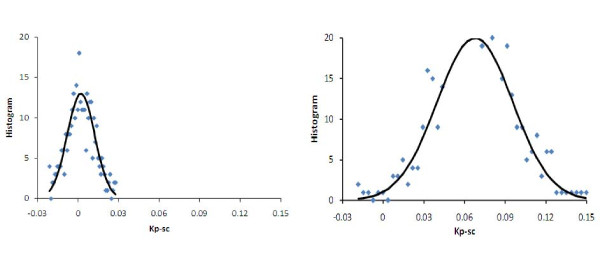
**Histograms of *K*_*p*-*sc*_-maps from a normal caudal section (left) and epicenter of the injury (right)**. Solid lines are fit to a Gaussian function. A threshold of 0.025 min^-1 ^was determined from the histogram on the left. This value was used for segmenting the regions with compromised BSCB permeability in the maps from the other slices.

**Figure 11:**
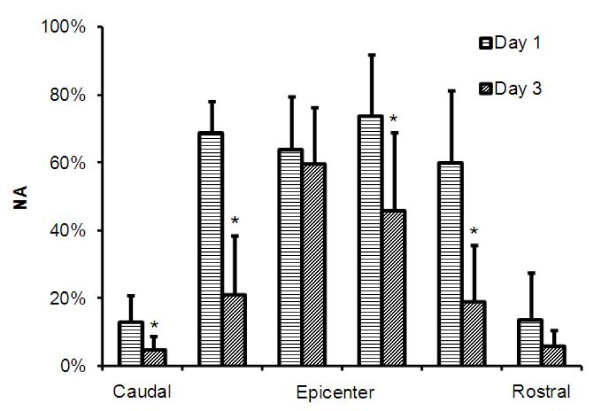
**Normalized area (mean ± std) measurements from 6 slices centered at the epicenter from all animals on day 1 and day 3**. * denotes statistically significant difference (P < 0.05).

**Figure 12:**
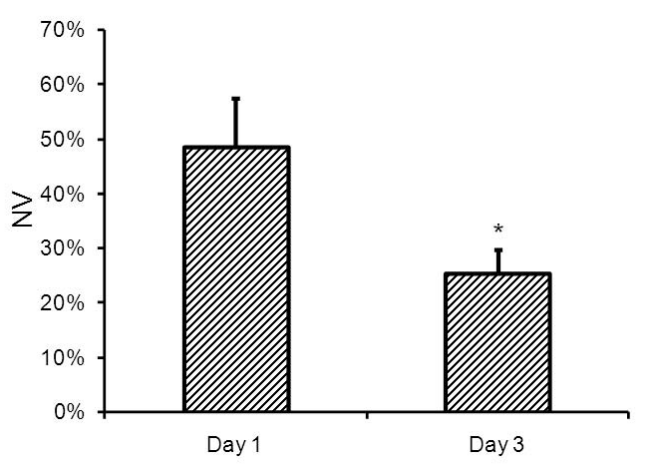
**Normalized volume (mean ± std) measurements from all animals on day 1 and day 3**. * denotes statistically significant difference (P < 0.05).

The area and volume of the regions with damaged BSCB were measured semi-automatically as described above. Figure 10 shows two histograms of *K*_*p*-*sc *_maps from a normal section and epicenter. The histogram from the normal section was used to determine a threshold for spatially segmenting the *K*_*p*-*sc *_maps to measure the areas in the other slices. The inter- and intra-variations for the threshold values estimated on days 1 and day 3 were within the range 0.03 ± 0.01 min^-1^. Figure 11 compares *NA *measurements from *K*_*p*-*sc *_maps of 6 slices centered at the lesion from all animals. These results indicated that four slices (in 4 mm space) around the epicenter suffered the most compromise in BSCB, covering about 70% of the total cross section of the SC parenchyma on day 1. But, on day 3, the regions with damaged BSCB were reduced in size to about 55% for the two middle and 20% for the remaining two slices. Such behavior indicated that the spatial distribution of the areas with compromised BSCB shrunk with time. This was consistent with the previous reports, where the lesion size shrank with time in directions both across and along the cord, which was a unique behavior seen in mice SCI [27,28]. This outcome was further supported by the normalized volume (*NV*) measurements that decreased from day 1 to 3, as shown in Figure 12. The *NV *data from all animals indicated that volume of BSCB breakdown occupied 48% of the total volume of the SC on day 1 as compared to 25% on day 3. The difference between the two measured volumes was statistically significant. These results from day 1 and day 3 together implied that dynamic remodeling of the BSCB permeability took place as part of the ongoing neurovascular repair and recovery processes in injured cord.

**Figure 13:**
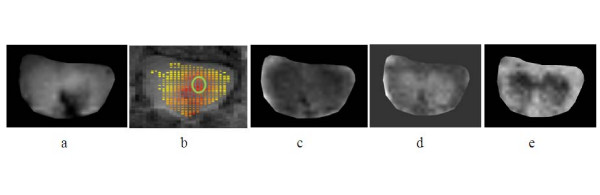
**Postinjury day 1**: a) anatomical T2-weighted image of an injured SC, b) *K*_*p*-*sc *_map, c) λ_|| _map, d) λ_⊥ _map and e) *FA *map. Green circle denotes the region of interest chosen within the GM to compute the mean values for the parameters *K*_*p*-*sc*_, λ_||_, λ_⊥ _and *FA *from the corresponding maps.

Figure 13 shows anatomical T2-weighted image of an injured SC and the computed maps (*K*_*p*-*sc*_, λ_||_, λ_⊥ _and FA) from the same slice orientation. The green circle plotted on the *K*_*p*-*sc *_map represents a region of interest with increased BSCB permeability within the GM. The mean values for the parameters *K*_*p*-*sc*_, λ_||_, λ_⊥ _and *FA *residing within such selected regions were computed for each animal. The results from all animals were further averaged and compared in Table 1. The differences between the measurements on days 1 and 3 were found to be statistically significant (P < 0.05). Normative λ_|| _and λ_⊥ _measurements from GM of normal mouse SC (λ_|| _= 1.25 × 10^-3 ^mm^2^/s, λ_⊥ _= 0.47 × 10^-3 ^mm^2^/s and *FA *= 0.61) were reported earlier [21]. Comparing these results with the corresponding ones in the table indicates significant differences, demonstrating the degree of sensitivity of these parameters to the neuropathology in injured cords. The data in the table also shows that the parameters *K*_*p*-*sc*_, λ_|| _and *FA *decrease, but λ_⊥ _increases from day 1 to day 3. Such trends are suggestive of vascular restoration, further disruption of axonal integrity, loss of anisotropy and increased demyelination within the injured SC as time progressed.

**Table 1:** BSCB permeability and microstructural measurements on day 1 and day 3 (mean ± standard deviation). *P *values for the measurements between day 1 and 3 are given in the last row.

	*K*_*p*-*sc *_(min^-1^)	λ_|| _(×10^-3 ^mm^2^/s)	λ⊥ (×10^-3 ^mm^2^/s)	*FA*
Day 1	0.123 ± 0.008	0.788 ± 0.022	0.579 ± 0.016	0.183 ± 0.025

Day 3	0.076 ± 0.006	0.694 ± 0.026	0.604 ± 0.015	0.097 ± 0.021

*P*	0.002	0.001	0.002	0.006

The level of the one-to-one dependencies between the parameter *K*_*p*-*sc *_and the measurements λ_||_, λ_⊥ _and *FA *are given in Table 2. From the values in the table, negative correlations were found between *K*_*p*-*sc *_and λ_|| _and between *K*_*p*-*sc *_and *FA*. *K*_*p*-*sc *_and λ_⊥ _were also correlated, but positively. Combining these results with the histological findings (Figures 7, 8 and 9) suggested that a strong relationship exists between vascular integrity and the structural state of the neuronal tissue as assessed by the diffusion measurements. These results were in line with the prior knowledge that more vascular damage is likely to cause greater neuronal loss following SCI.

**Table 2:** Pearson's correlation coefficient results between the BSCB permeability and microstructural measurements on day 1 and day 3.

	ρ(*K*_*p*-*sc*_, λ_||_)	ρ(*K*_*p*-*sc*_,λ_⊥_)	ρ(*K*_*p*-*sc*_, *FA*)
Day 1	-0.87	0.64	-0.77

Day 3	-0.92	0.97	-0.70

## Discussion

**Figure 14:**
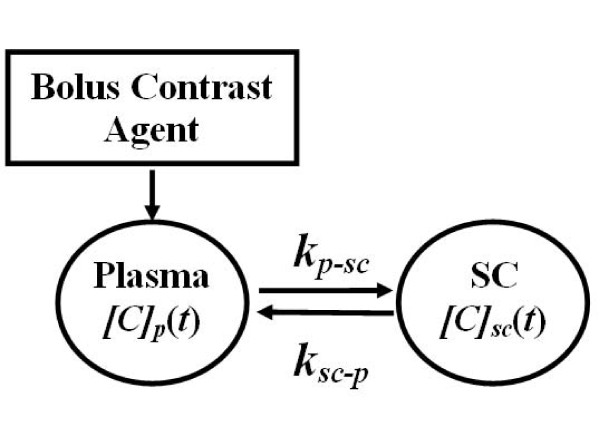
**Two-compartment pharmocokinetic model representing the distribution of the contrast agent in plasma and injured spinal cord tissue**. The subscripts *p *and *sc *denote plasma and spinal cord. *[C]*_*p*_(*t*) and *[C]*_*sc*_(*t*) are time-dependent concentrations in mmol/L in plasma and spinal cord compartments, respectively. The parameters *k*_*p*-*sc *_and *k*_*sc*-*p *_denote transfer rate constants in l/min for forward transfer of the contrast agent from plasma-to-spinal cord and reverse transfer from spinal cord-to-plasma compartments, respectively.

Preclinical neuroimaging methods are required for evaluating the degree of initial mechanical damage and for monitoring the subsequent secondary events following SCI in experimental research. With this purpose in mind, we and others have investigated a variety of MRI methods to obtain anatomical, vascular and structural information from the injured SC [14,21,29-39]. However, to date, no study has combined DCE-MRI and DTI protocols to simultaneously evaluate the microvascular and microstructural changes in injured SC. The current study is the first to jointly apply these techniques, along with anatomical imaging, to evaluate SCI in mouse. An additional achievement that distinguishes this study from the previous works is the computational algorithm implemented for quantitatively mapping the spatial distribution of BSCB permeability in injured SC. This algorithm was derived from a pharmacokinetic model (Figure 14) that was originally developed to represent plasma and injured SC by two compartments, and to determine the exchange of the contrast agent in between [6]. In this model, immediately following the IV bolus injection, the transport of the contrast agent was considered unidirectional from plasma to the injured SC tissue. This feature allowed associating the barrier permeability with the transfer rate constant from plasma-to-spinal cord. Such association was also proved to be critical for simplifying the computational analysis whereby requiring only the early part of the relative intensity enhancement (Figure 4). This ultimately led to the estimation of localized changes in the barrier dysfunction throughout the SC (Figure 5).

Our MRI-based data, as in Figures 3, 4 and 5, and histological analysis, as in Figures 7, 8 and 9, demonstrated the dynamic remodeling of the BSCB as part of the ongoing repair and recovery processes in the injured SC tissue. Using postmortem analysis in mouse, Whetstone et al. [2] reported that SCI results in a biphasic, temporal pattern of barrier leakage to tracer – luciferase. In their study, the barrier leakage was shown to extend beyond the epicenter into segments that were within 6 mm rostral and caudal to the epicenter. The leakage was seen to be mostly pronounced within the first 35 min after the injury followed by a gradual decline within the first 24 hr. A second peak of abnormal barrier permeability was reported at 3 days after the injury, which significantly exceeded that observed at 24 hr. Regarding the barrier permeability to the contrast agent, we obtained comparable results that showed stronger intensity enhancement on day 3 than day 1. This agreement is encouraging and warrants further exploration, such as investigation of the loss of blood vessels and revascularization in injured mouse SC using DCE-MRI in conjunction with MR angiography modality [5,32,40].

Requiring only the initial portion of the intensity enhancement to assess barrier function offers benefits in terms of reducing the imaging time substantially. In the current study, we acquired T1-weighted images using a spin-echo sequence which required relatively long acquisition time. This is one limitation of the approach. Higher temporal resolution can be achieved between the precontrast and postconrast acquisitions by employing faster imaging sequences. Such ability should further improve the time and accuracy of estimating the barrier permeability. For this purpose, echo-planar imaging appears promising [41].

From the application point of view, providing information about the barrier function should be useful in at least three circumstances. One situation is in characterizing the SCI model where the resulting barrier leakage in a particular SCI model may be another parameter to be defined, and referred back when needed to check against the consistency of the newly induced injuries. This would enable the confirmation of whether later injuries have similar properties in terms of the vascular response. Traditional histological analysis was employed to understand the sensitivity and specificity of the barrier damage to graded mechanical perturbations [3]. Our approach offers an efficient alternative and may become a preferred choice for such purpose. In the second case, quantitatively evaluating the severity of the barrier damage as soon as possible after the injury may provide early indications of the significance and level of the expected secondary processes that are likely to increase neuronal loss. Such capability can have significant prognostic value. Figure 13 and comparative analysis in Tables 1 and 2 clearly provided evidence that correlations exist between DCE-MRI and DTI based measurements. This has important implications in practice, especially when the examined SCI produces poor quality of DTI acquisition or the analysis of the resulting data is not feasible. In such challenging situations, including the DCE-MRI protocol in the scan may supply information on the condition of the vasculature, which may indirectly help to determine the level of neuronal damage in the underlying SC tissue by using the close associations demonstrated in Table 2. Lastly, monitoring the barrier function may have important implications in predicting the efficacy of targeted drug or gene delivery through the permeable barriers when potential therapeutic interventions are considered. While this aspect is foreseeably achievable if the drug or transfection vector has molecular weight comparable to that of the contrast agent used in this study, it remains to be seen if the approach would be viable in cases when the drug or gene has significantly larger size.

## Conclusion

This study has demonstrated the potential of DCE-MRI method to assess the BSCB dysfunction in injured spinal cord noninvasively. The method involved acquiring only two T1-weighted images; precontrast and postcontrast, acquired with a minimum time delay following the injection of contrast agent. This simple data acquisition strategy provides sufficient temporal information on the contrast-enhancement that was necessary to map the spatial distribution of BSCB permeability. The BSCB dysfunction correlated strongly with the degree of axonal loss and demyelination. The relationship between the vascular damage and neuronal loss in injured SC has been well-established previously, but by using histological techniques. Reconfirming this association using DCE-MRI and DTI data acquired from living animals has important implications in translational research. Preclinical efforts are currently focused on developing BSCB permeable drugs for improving neurovascular function by repairing vascular network, delaying neurodegenerative processes or promoting neuronal recovery. Research efforts are also underway for understanding the role of specific genes in vascular reorganization, neuronal repair and recovery from an injury on experimental test systems involving different strains of "transgenic" or gene knock-out mice. On the basis of multiple neuroimaging methodologies developed in this study, scanning the same animal model with anatomical, DCE-MRI and DTI protocols provides measurable parameters that can serve as sensitive and specific in vivo neurovascular biomarkers for comprehensively evaluating the pathological state of the injured SC, may offer a prognostic value for functional recovery from SCI and potentially serve as a monitoring tool for evaluating the efficacy of a treatment efficacy.

## Competing interests

The authors declare that they have no competing interests.

## Authors' contributions

IT performed the statistical analysis. PCC postprocessed the acquired DCE-MRI data. HES contributed with the histology and staining. MMD evaluated the pathology. MB was responsible for the conception and design of the experiments as well as the analysis and interpretation of the data collectively. All authors read and approved the final manuscript.

## Appendix

The presence of paramagnetic contrast agent at concentration *[C] *(in *mmol*/*L*) in neuronal tissue of an injured SC was detected by relative intensity enhancement (*RIE*) on T1-weighted magnetic resonance images. Postcontrast *RIE *at time *t *was calculated for each pixel at a spatial position (*x*, *y*) and a slice location *z *using the formula(1)

Here, *I*(*t *= 0, *x*, *y*, *z*) and *I*(*t*, *x*, *y*, *z*) represent intensities in the precontrast and *t*^th ^postcontrast images of a given slice *z*, respectively. The mask *Mask*(*z*) was obtained by using manual segmentation applied on the postcontrast images where the SC was delineated best. The mask covered the cord but excluded cerebrospinal fluid, which also exhibited postcontrast enhancement. The total cross-sectional area of the SC at that particular slice was measured by the area covered by the mask.

When a spin-echo sequence is used for DCE-MRI acquisition and a small dose of contrast agent is delivered as a bolus, as in this study, the SC concentration *[C]*_*sc *_and *RIE *are related by the formula [4](2)

Here, *T*_10 _denotes relaxation time in second (s) before administering the contrast agent and *r*_1 _is relaxivity introduced by the contrast agent to that of the underlying SC tissue and expressed in *mM*^-1 ^*s*^-1^. For mice scanned at 9.4 T, *T*_10 _was reported to be 1730 ms for the gray and 1690 ms for the white matters of the SC [21], but *r*_1 _associated with the contrast agent for these tissue types has yet to be determined.

Equation [2] plays an important role when constructing a pharmacokinetic model to theoretically represent the uptake of contrast agent in an injured SC [4]. Construction of a proper pharmacokinetic model requires at least two compartments – one for the plasma and one for the cord, as shown in Figure 1[6]. In this model, two rate constants (*min*^-1^) quantitatively describe the transport of contrast agent from plasma-to-SC and SC-to-plasma. Following an IV bolus injection, the contrast agent first leaks from plasma to the injured SC tissue through the compromised BSCBs. This thereby allows the representation of the barrier permeability with the rate constant *k*_*p*-*sc *_governing the unidirectional transfer of the contrast agent from plasma-to-SC. This rate constant is then estimated using the formula [4,6]. In this expression, *[C]*_*p*_(*t *= 0) denotes the initial concentration of the contrast agent in mouse plasma and computed from the injected dose and the total plasma volume. The operator  denotes time derivative evaluated immediately after the delivery of the contrast agent. Using Eq. [2], we can write(3)

Considering that the factor *T*_10_*r*_1 _has negligible dependence on spatial location, we reorganize Eq. [3] to obtain a new parameter that is linearly proportional to *k*_*p*-*sc*_:(4)

This equation indicates that the initial slope of *REI *provides a measurable quantity representing the BSCB permeability at a spatial location (*x, y, z*). Specifically, numerical computation of Eq. [4] involves discrete derivative operation  for *t *= 0 and Δ*T *= 10 min – the temporal resolution in our DCE-MRI acquisitions. By noticing that *RIE *(*t *= 0, *x*, *y*, *z*) = 0, the derivative operation can be simplified further. In this study,  was calculated for each *x*, *y *∈ *Mask*(*z*) and the results were put together to form a 2-D quantitative *K*_*p*-*sc *_map delineating the regions of the injured SC with compromised BSCB permeability for each slice.

## Pre-publication history

The pre-publication history for this paper can be accessed here:

http://www.biomedcentral.com/1471-2342/9/10/prepub
